# Exploring seascape genetics and kinship in the reef sponge *Stylissa carteri* in the Red Sea

**DOI:** 10.1002/ece3.1511

**Published:** 2015-06-01

**Authors:** Emily C Giles, Pablo Saenz-Agudelo, Nigel E Hussey, Timothy Ravasi, Michael L Berumen

**Affiliations:** 1 Red Sea Research Center, King Abdullah University of Science and TechnologyThuwal, 23599-6900, Saudi Arabia; 2 Instituto de Ciencias Ambientales y Evolutivas, Universidad Austral de ChileValdivia, Chile; 3 GLIER, University of WindsorWindsor, Ontario, Canada; 4 Division of Biological and Environmental Sciences and Engineering, Division of Applied Mathematics and Computer Science, King Abdullah University of Science and TechnologyThuwal, 23599-6900, Saudi Arabia

**Keywords:** Environmental gradient, isolation by distance, isolation by environment, porifera, relatedness, seascape genetics

## Abstract

A main goal of population geneticists is to study patterns of gene flow to gain a better understanding of the population structure in a given organism. To date most efforts have been focused on studying gene flow at either broad scales to identify barriers to gene flow and isolation by distance or at fine spatial scales in order to gain inferences regarding reproduction and local dispersal. Few studies have measured connectivity at multiple spatial scales and have utilized novel tools to test the influence of both environment and geography on shaping gene flow in an organism. Here a seascape genetics approach was used to gain insight regarding geographic and ecological barriers to gene flow of a common reef sponge, *Stylissa carteri* in the Red Sea. Furthermore, a small-scale (<1 km) analysis was also conducted to infer reproductive potential in this organism. At the broad scale, we found that sponge connectivity is not structured by geography alone, but rather, genetic isolation in the southern Red Sea correlates strongly with environmental heterogeneity. At the scale of a 50-m transect, spatial autocorrelation analyses and estimates of full-siblings revealed that there is no deviation from random mating. However, at slightly larger scales (100–200 m) encompassing multiple transects at a given site, a greater proportion of full-siblings was found within sites versus among sites in a given location suggesting that mating and/or dispersal are constrained to some extent at this spatial scale. This study adds to the growing body of literature suggesting that environmental and ecological variables play a major role in the genetic structure of marine invertebrate populations.

## Introduction

Historically, gene flow in the marine realm was thought to generate panmictic populations semiconnected ones following a stepping stone trajectory, or completely isolated groups. However, it is now widely accepted that genetic connectivity can take any combination of the above-mentioned trajectories and can differ given the temporal and spatial scale at which it is investigated (Selkoe et al. 2008). It is thus noted that conducting population genetics studies at various temporal and spatial scales can yield a more accurate picture of the genetic distribution of a species (Wiens 1989; Levin 1992).

Studies conducted at various geographic scales are useful to determine the importance of spatial heterogeneity on shaping genetic connectivity. For example, many marine studies have explored the effect of increasing spatial distance on gene flow (Isolation by Distance, IBD) (reviewed in Hellberg 2007; Jones et al. 2009; Selkoe and Toonen 2011), but the direct relationship between geographic distance and genetic distance is not always linear. Often, correlations are weak and significant only due to large sample sizes (as explained by Jenkins et al. 2010), and IBD relationships do not always hold at multiple spatial scales. Processes operating at one spatial scale can influence recruitment, survival, and admixture in ways that go undetected if a study is conducted at a larger spatial scale (Manel and Holderegger 2013). More recently, this has been shown to occur frequently with invertebrate populations (Gorospe and Karl 2013; Ordóñez et al. 2013). Many abiotic and biotic factors are known to influence marine invertebrate population dynamics and these factors operate at diverse spatial scales (see review by Sanford and Kelly 2011). Temperature, light, and salinity gradients exist at fine vertical scales to large latitudinal scales. Gradients in primary productivity, nutrient availability, and predator abundance can exist at yet other scales (see review by Sanford and Kelly 2011). Thus, the combination of the various scales of factors that influence marine invertebrates yields population contingencies that are much more complex than those resulting from genetic drift alone.

Recently, a metric known as Isolation by Environment (IBE) has been utilized to analyze the effect of increasing environmental heterogeneity on genetic structure (e.g., Selkoe et al. 2008; Nanninga et al. 2014; Wang and Bradburd 2014). While analyzing the influence of environmental factors driving genetic variation among populations is not as common as simply testing for IBD, the few studies that have tested for IBE show the importance of considering both geography and environment before drawing conclusions regarding barriers to population connectivity (e.g., Crispo et al. 2006; Cushman et al. 2006; Wang and Summers 2010; Lee and Mitchell-Olds 2011; Wang 2013; Nanninga et al. 2014). Most notably at large scales, environmental heterogeneity can promote genetic divergence, which could impact juvenile settlement, larval survival, and mortality, as well as promote potential differences in reproductive success (Selkoe et al. 2006, 2008; Schmidt et al. 2008). Environment has been shown to be particularly important in segregating long-lived sessile organisms that have wide-range dispersal (Goreau 1959; Kinzie 1973; Paine et al. 2009; Baldeck et al. 2013). Further to this, transplant experiments have shown that mature organisms experience higher mortality in non-native habitats (Marshall et al. 2010; Prada and Hellberg 2013) and local adaptation has been shown to reach 71% in some plants (Leimu and Fischer 2008). Yet, despite the fact that sponges are both long living and sessile, the extent to which environmental condition promotes local adaptation in phylum Porifera is yet untested.

Sponges are among the most ancient metazoans making them target organisms for many evolutionary studies (e.g., Li 1998; Srivastava et al. 2010), yet to date, only approximately ten population genetics studies conducted at ecologically relevant time scales (e.g., using highly variable microsatellite markers) have been published (reviewed in Uriz and Turon 2012). Several of these studies confirm the inherent complexity of sponge population biology as four of these studies have reported strong genetic structure at small spatial scales (<1 km) (Duran et al. 2004; Blanquer et al. 2009; Blanquer and Uriz 2010; Guardiola et al. 2011), one study found genetic differentiation even at the scale of tens of centimeters (Calderón et al. 2007), and yet other studies found genetic structure at large spatial scales (>100 km) (Dailianis et al. 2011; Chaves-Fonnegra et al. 2015). These studies and others conducted using mitochondrial and nuclear markers (e.g., DeBiasse et al. 2009; López-Legentil and Pawlik 2009) suggest that while dispersal is limited in some species, there is no general pattern of strict isolation, isolation by distance, or panmixia in phylum Porifera.

The aim of this study was to assess the broad and fine-scale population genetic structure of a common Indo-Pacific reef sponge, *Stylissa carteri*, and determine factors that influence population differentiation. As a member of the order Halichondrida, *Stylissa carteri* is thought to have indirect and internal reproduction with a parenchymella type I larvae, although gametogenesis is yet unstudied in the genus *Stylissa* (Maldonado 2006). Members of phylum Porifera are thought to disperse through an often-short pelagic larval phase (Uriz et al. 1998), and once settled, the sessile adult sponge is dependent on the constraints of its surrounding environment in terms of food availability (Lesser 2006), reproductive potential (Maldonado and Young 1996), and predation (Pawlik et al. 2013). At the broad scale, genetic connectivity was explored using Bayesian clustering, IBD, and IBE analyses to determine whether barriers to gene flow are due in part to geographic and/or environmental factors. At finer scales, we investigated the presence of limited larval dispersal and/or nonrandom mating by evaluating spatial autocorrelation analysis within 50-m transects and comparing the proportion of full-siblings among individuals in a hierarchical design (transect/site/location). Taken together, the results gathered yield valuable information regarding the importance of environmental heterogeneity in the Red Sea and how this heterogeneity predicts gene flow and potentially local adaptation in a sessile marine organism.

## Materials and Methods

### Study system

**Figure 1:**
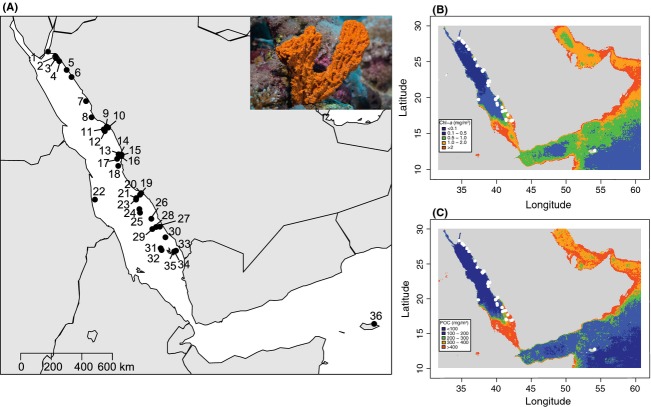
(A) Sampling sites in the Red Sea and northwest Indian Ocean. Photograph of *Stylissa carteri* from the Red Sea (Photograph credit: Tane Sinclair-Taylor). Contour maps of 9-year averaged Red Sea chlorophyll (B) and particulate organic carbon (C). More information can be found in Tables S1 and S2.

The Red Sea is an understudied system (Berumen et al. 2013) known for its high endemism (14–50% depending on the taxa see Roberts et al. 1992; Randall 1994; Cox and Moore 2000; DiBattista et al. 2013), its extreme salinity (37–42‰), and temperature ranges (20–32°C) (Raitsos et al. 2011). Extending from 30°N to 12.5°N, the Red Sea is marked by a latitudinal environmental gradient in primary productivity and turbidity, both increasing in southern waters (Raitsos et al. 2013; Fig.1). The study subject, *Stylissa carteri*, has a widespread Indo-Pacific distribution (Hooper and Van Soest 2004) and is abundant in coastal Red Sea waters (with the exception of the Gulf of Aqaba) typically between 5 and 15 m in depth (E. C. Giles, unpubl. data).

### Sample collection

Sponge tissue samples (n = 966) were collected by SCUBA between October 2012 and February 2014 from a total of 36 reef sites. A total of 34 of those sites span 1500 km of Saudi Arabian coastline, one is off the coast of Sudan, and one is off the coast of Socotra in the Indian Ocean (Fig.1, Table1). Approximately 1 g of tissue was cut from each sponge and stored in 96% ethanol until further analysis was undertaken. Select samples were analyzed by traditional taxonomy and verified as *Stylissa carteri* by two sponge taxonomic experts (Lisa Goudie and Nicole de Voogd).

**Table 1:** Summary statistics for *Stylissa carteri* listed from northernmost to southernmost sampled sites (Site Number, Location, Site Name, ^1^fine-scale sampling was conducted, GPS). The number of samples amplified (*N*), allelic diversity (*N*_A_), observed heterozygosity (*H*_O_), unbiased expected heterozygosity (*uH*_E_), and fixation index (*F*_IS_) are given for each population. Values are averaged over 9 loci

Site number	Location	Site name	GPS coordinates	*N*	*N*_A_	*H*_O_	*H*_E_	*F*_IS_
1	North	Gulf of Aqaba	28.185, 34.638	7	3.111	0.313	0.485	0.3743*
2	North	Jazirat Burcan	27.910, 35.065	14	4.556	0.452	0.533	0.1565
3	North	Shi’b Pelam	27.817, 35.107	11	3.889	0.364	0.499	0.2807
4	North	Jaz’air Sila	27.638, 35.306	7	4	0.5	0.59	0.1663**
5	North	An Numan	27.139, 35.751	20	4.556	0.389	0.528	0.2703**
6	North	Ras Al-Ubayd	26.736, 36.044	12	4.333	0.426	0.544	0.2251**
7	North	Shaybarah	25.362, 36.913	8	3.222	0.361	0.46	0.2272
8	Yanbu	Marker9	24.443, 37.248	11	4.556	0.444	0.625	0.2988**
9	Yanbu	Reef2^1^	23.907, 38.153	44	5.889	0.42	0.59	0.2902**
10	Yanbu	Refinery^1^	23.853, 38.240	54	6.333	0.39	0.538	0.2765**
11	Yanbu	7Sisters	23.753, 37.974	24	5.889	0.349	0.595	0.4194**
12	Yanbu	Tistis	23.651, 38.035	22	4.889	0.313	0.546	0.4322**
13	Thuwal	Shib Nazar^1^	22.331, 38.863	72	6.889	0.38	0.564	0.3245**
14	Thuwal	Al Fahal	22.312, 38.978	22	5.444	0.399	0.549	0.2769**
15	Thuwal	Abu Shoosha	22.305, 39.049	24	6.444	0.416	0.597	0.3065**
16	Thuwal	Fsar	22.227, 39.030	24	5.667	0.446	0.533	0.1636**
17	Thuwal	Abu Madafi^1^	22.074, 38.778	24	5	0.42	0.522	0.2014**
18	Jeddah	Obhur	21.671, 38.844	20	5.111	0.38	0.597	0.3665**
19	Al-Lith	Whale Shark Reef^1^	20.119, 40.220	71	6.111	0.375	0.553	0.3207**
20	Al-Lith	Reef3^1^	20.031, 40.147	46	6.222	0.385	0.549	0.2977**
21	Al-Lith	MarMar^1^	19.838, 39.921	27	5.667	0.354	0.548	0.3495**
22	Sudan	Sanganeb	19.753, 37.448	11	4.111	0.392	0.525	0.2671**
23	Al-Lith	Malatu^1^	19.752, 39.910	57	6.556	0.365	0.522	0.3013**
24	Farasan Banks	Abu Dauqa	19.209, 40.109	15	4.667	0.413	0.525	0.2182*
25	Farasan Banks	Dolphen Lagoon	19.005, 40.148	12	4.333	0.354	0.465	0.2488**
26	Farasan Banks	Ablo Island	18.660, 40.827	19	4.667	0.365	0.516	0.2976**
27	Farasan Banks	Marka Island	18.209, 41.335	27	4.778	0.436	0.575	0.2465**
28	Farasan Banks	Atlantis Shoal	18.189, 41.111	16	5.556	0.502	0.621	0.1943*
29	Farasan Banks	Shib Radib	18.073, 40.886	44	6.667	0.393	0.538	0.2729**
30	Wassalyat Shoals	Mamali Kabir	17.605, 41.671	37	5.556	0.405	0.577	0.3005**
31	Farasan Islands	Baghlah	16.980, 41.385	9	3.778	0.444	0.519	0.1504
32	Farasan Islands	Dhi Dahaya	16.875, 41.440	28	5.667	0.376	0.551	0.3178**
33	Farasan Islands	Zahrat Durakah	16.840, 42.305	24	4.889	0.416	0.522	0.2031**
34	Farasan Islands	Abulad Island	16.798, 42.199	46	5.778	0.43	0.572	0.25**
35	Farasan Islands	Tiger Head	16.791, 42.199	29	4.889	0.441	0.55	0.2013**
36	Soccotra	Soccotra	12.670, 54.178	12	3.222	0.367	0.426	0.1442

Significant (*F*_IS_) values are indicated

* *P *<* *0.05

** *P* < 0.01 (FDR correction).

At eight of the 36 reef sites, sampling was conducted to determine fine-scale structure patterns both within and among sites. At each of three locations (Yanbu, Thuwal, and Al-Lith), transects were sampled in different sites (at Yanbu and Thuwal, two sites yielding four transects were sampled, and at Al-Lith, four sites yielding eight transects were sampled). Sites were separated by no more than 30 km, 38 km, and 52 km at Yanbu, Thuwal, and Al-Lith, respectively. At each of the eight sites (Site 9, Site 10, Site 13, Site 17, Site 19, Site 20, Site 21, Site 23, indicated in Table1), two belt transects 50 m x 4 m were laid on the leeward side of the reef and an effort was made to sample all *Stylissa carteri* individuals within each transect. As individuals were sampled, their depth and location within the transect were recorded.

### Genetic analysis

The DNA was extracted from 200 to 250 mg of sponge tissue with cell lysis immersion in 600 *μ*L of 1% 2-mercaptoethanol RLT buffer (Qiagen, Germany) and shaking incubation or overnight proteinase K incubation at 56°C (Qiagen). Following cell lysis, the DNA extraction method proceeded via the Allprep DNA/RNA mini Kit (Qiagen).

Nine microsatellite markers were used. Marker details and PCR parameters are specified in Giles et al. (2013) and summarized in Table S1. The microsatellite markers were combined into five multiplex mixes based on fragment sizes and fluorescent dye tags. For loci that did not amplify at first, PCRs were redone without multiplexing. Positive PCR products were diluted with Hi-Di formamide (Applied Biosystems) and GeneScan 500-LIZ size standard (Applied Biosystems, CA, USA) and run on an ABI 3730xl genetic analyzer (Applied Biosystems) for fragment size identification. All samples with more than one missing loci were removed from the dataset.

Samples were genotyped using the software GENEMAPPER 4.0 (Applied Biosystems). Allelic frequencies, number of alleles (*N*_A_), observed (*H*_O_), and unbiased expected heterozygosities (u*H*_E_) were estimated for each site using the software Genalex (v6.5; Peakall and Smouse 2012). Calculations were made for the inbreeding coefficient (*F*_IS_), and tests for linkage disequilibrium (LD) and deviations from Hardy–Weinberg proportions (HWE) were determined using Genepop (Raymond and Rousset 1995; Rousset 2008). Tests for significant deviations from LD and HWE were made using permutations via Markov chain reshuffling (10,000 dememorizations, 1000 batches, and 10,000 iterations per batch). Corrections for multiple testing were made using false discovery rate (FDR, Benjamini and Hochberg 1995). Furthermore, the data were tested for the presence of null alleles using MICROCHECKER (v2.2.3; Van Oosterhout et al. 2004).

Clonality within the entire dataset was determined by measuring the number of 100% multilocus matches (Genalex v6.5). To avoid over-representation of individual genotypes, one of each of the members of a clonal pair was removed from the dataset for subsequent genetic analyses.

### Broad-scale structure

Bayesian clustering via the program STRUCTURE (v2.3.4 Pritchard et al. 2000) was employed to ascertain the most likely number of populations (K-clusters). An admixture model was used with sampling location as a prior. Allele frequencies were assumed to be correlated among populations. The model was run for values of K from 1 to 20. For each K, the model was run 10 times with a burn-in step of 300,000 iterations and 500,000 subsequent iterations. STRUCTURE Harvester (Earl and vonHoldt 2012) was used to determine which K best describes the data according to the highest averaged maximum-likelihood score and Evanno’s delta K.

We also examined population genetic differentiation via the analysis of molecular variance (AMOVA) using the program Genalex. The AMOVA was run four times, once without regional classification of the samples, once with a two-region classification, once with a three-region classification, and once with a four-region classification. The regional classifications were made based on results from the STRUCTURE (v2.3.4 Pritchard et al. 2000) analysis and based on the knowledge that Socotra represents a distant and potentially unique population as samples from this site are the only ones which were taken outside of the Red Sea. Thus, the two-region classification scheme included sites 1–32 and 36 in one region and sites 33–35 in the second region. The three-region classification scheme included sites 1–32 in the first region, sites 33–35 in the second region, and Site 36 as the third region. The four-region classification scheme included sites 1–29 and 31–32 in one region, Site 30 as a region, sites 33–35 as a region, and Site 36 as a region (see Table1 for site information). Significant pairwise *F*_ST_ comparisons were determined based on FDR. Finally, pairwise population *F*_ST_ and *G*_ST_ estimates were calculated in Genalex using the same AMOVA framework with 9999 permutations to test for significance. Pairwise *F*_ST_ and *G*_ST_ values were calculated for the 36 pairwise comparisons; pairwise values for Site 36 were calculated using only eight loci as one loci would not amplify for the Socotra samples.

### Geographic analysis

Pairwise geographic distance (Euclidean distance in km) was calculated between each of the 35 sample sites following Nanninga et al. 2014. To investigate the determinants of genetic distance in the Red Sea, the Socotra data was withheld from all subsequent analysis as (1) this site is outside the area of primary interest; (2) it is geographically isolated from all the other sites; and (3) it appears to be very distinct (highest pairwise *F*_ST_ are with this site) from the rest of the dataset, probably due to its geographic isolation. Thus, the inclusion of this disparate site could confound meaningful results about processes within the Red Sea.

Using a Mantel test with 9999 permutations, pairwise matrices of linearized *F*_ST_ (*F*_ST_ / (1 − *F*_ST_)) and geographic distance were compared to determine the degree of isolation by distance (IBD) of *S. carteri* populations. Pearson correlation coefficients were calculated for the linear relationship between geographic distance and genetic distance. Regressions were made twice, once using raw matrices and secondly on matrices standardized by subtracting the mean and dividing by the standard deviation of the dataset.

### Environmental analysis

Environmental data were gathered from the NASA Giovanni website using the MODIS-Aqua 4 km database (http://oceancolor.gsfc.nasa.gov) with standard NASA estimate algorithms. Color radiometry measurements of sea surface temperature (day DSST °C and night NSST °C), colored dissolved organic matter (CDOM), chlorophyll a (CHLA mg/m^3^), and particulate organic carbon (POC mg/m^3^) were used as the environmental criteria in this study. All criteria for the 9-year winter season (October 2003 to May 2012) and annual (January 2003 to December 2012) time frame were averaged and downloaded yielding a value of SST, CDOM, CHLA, and POC for the entire Red Sea and northwest Indian Ocean for winter and annual means. Due to seasonal cloud cover/shallow sea level, there was a lack of satellite data for summer months; thus, this time frame was excluded from the study. The data used for the environmental analysis were selected because they are the most robust available measurements of large-scale environmental criteria for the Red Sea. The raw data for the five environmental criteria assessed were averaged because it is thought that processes such as reproduction, dispersal, and ultimately gene flow happen over demographic to evolutionary time scales. Contour maps of the 9-year annual averages of CHLA and POC for the entire Red Sea and northwest Indiana Ocean were made in R using the raw data downloaded from MODIS-Aqua (Fig.1).

These datasets were then mined for the values that corresponded to the point (4 km^2^ resolution) nearest to the 36 sample site locations in this study (Table S2). Pairwise environmental distance (Euclidean distance in km) was calculated between each of the 35 sample sites located within the Red Sea following Nanninga et al. 2014 for all environmental criteria and the two time periods in question. Using Mantel tests with 9999 permutations, pairwise matrices of linearized *F*_ST_ (*F*_ST_ / (1 − *F*_ST_)) and environmental distance were compared to determine the degree of isolation by environment (IBE) of *S. carteri* populations. Pearson correlation coefficients were calculated for the linear relationship between environmental distance and genetic distance and significance tests were made (*α *= 0.05).

### Multiple matrix regression

Multiple matrix regression with randomization (MMRR) was used to determine which model best explained the observed trend in genetic distance (Wang 2013). The MMRR analysis was performed in R with 9999 permutations. The matrices used for this analysis were standardized by subtracting the mean and dividing by the standard deviation.

### Fine-scale structure

Genetic structure at the scale of 50 m was analyzed using the belt transect samples for eight of the 36 sample sites. Spatial autocorrelations generated in Genalex were used to test whether *S. carteri* multilocus genotypes were randomly distributed in space (within each transect) or not (following Smouse and Peakall 1999). Samples were binned into 5 m distance class sizes, and 999 permutations were used to determine the 95% confidence intervals around the null hypothesis of no spatial autocorrelation. All statistics were generated for each 50-m transect individually yielding two replicates for each of the eight reef sites.

Genetic structure at the scale of 50 m and at slightly larger scales (100–200 m depending on distance between 50-m transects) was also explored using the same belt transect samples in a hierarchical design. We used a likelihood-based approach implemented in KINGROUP v2 (Konovalov et al. 2004) to identify likely full-sibling pairs among all samples of each site. Two individuals were accepted as full-sibs if the LOD-score associated *P* value was ≤ 0.01. Significance testing was built based on the distribution of LOD scores from simulated 10,000 full-sibling pairs and unrelated pairs of individuals. The type II error associated with estimating the full-sibling pairs was estimated to be 34–35%. As a conservative measure, we chose to minimize type I error: that is, accept truly unrelated individual pairs as being full-sibs, at the expenses of type II error: rejecting pairs of individuals as being full-siblings when they truly are full-siblings. We estimated the proportion of full-siblings within transects, among transects within sites, and among sites within each location. We then compared the proportions of full-siblings: (1) within transects vs. among transects (within each site) and (2) within sites vs among sites (within each location) using a χ^2^ test of proportions conducted in R. We expected that if nonrandom mating or limited dispersal is occurring in a given location, the proportion of full-siblings within transects or within sites would be higher than the proportion of full-siblings among transects or among sites.

## Results

### Summary statistics

A total of 966 samples were genotyped at the nine microsatellite loci with the exception of the Socotra samples (*n* = 12) for which loci sc90 would not amplify. As the *S. carteri* microsatellite markers were developed on Red Sea samples, it is possible that a mutation in the primer-binding region of the more divergent Socotra samples is causing faulty amplification at this locus. The missing data of the Socotra samples did not affect the summary statistic analysis, as results were identical when analysis was made with and without loci sc90 and with and without the Socotra samples. In addition, of the 966 samples, 16 clonal pairs with identical multilocus genotypes were recovered (Table S3). Clones were more commonly found within sites (14 of the 16 pairwise comparisons) than among sites (2 of the 16 comparisons). One individual from each of the clonal pairs was removed from the dataset yielding a working dataset of 950 individuals with unique multilocus genotypes. All analysis was completed on the dataset with 950 individuals.

Overall, the data show a clear pattern of low heterozygosity (mean *H*_o_ = 0.399, mean *H*_e_ = 0.528), which was consistent at all sample site locations (Table1). This was met with a consistent and high inbreeding coefficient (*F*_IS_ ranged from 0.1442 to 0.4322) that was significant in 31 of the 36 sites. The allelic diversity ranged from 1 to 10 alleles per locus for any given site. While some deviations from HWE were found, no loci showed significant deviations (using FDR correction) from HWE at all sites tested (Table S4). Loci sc65 and sc88 showed no deviation from HWE, loci sc83 showed deviation from HWE in just two sites, and the remaining six loci showed deviations from HWE in approximately half of the sites tested. MICROCHECKER indicated the possible presence of null alleles in all loci as was to be expected with the observed heterozygote deficiencies. However, no loci showed the presence of null alleles at all sites. Null alleles were detected on average in 11 of 36 (99% CI) sites for a given locus. The lack of a clear pattern in deviations from HWE along with the findings of other studies that have shown reduced heterozygosities in sponge populations (Duran et al. 2004; Whalan et al. 2005; Duran and Rützler 2006; Guardiola et al. 2011; Chaves-Fonnegra et al. 2015) suggests that this is not a primer-binding artifact but rather a biological phenomenon that exists in sponge populations. Hence, these markers were considered suitable for the study at hand. Lastly, the tests for LD yielded zero significant values for linkage disequilibrium using FDR correction (1199 multiple comparisons).

### Broad-scale structure

**Figure 2:**
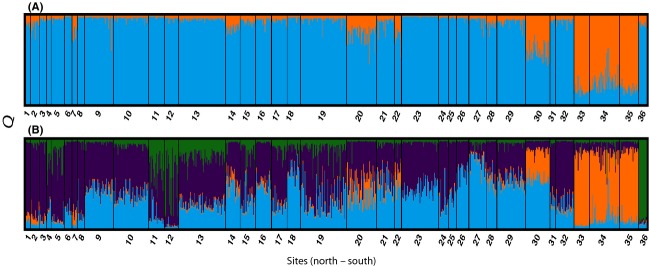
Large-scale population genetic differentiation inferred via Bayesian clustering. Prior information about the geographic origin of the samples was given. Best inferred clustering scheme was *K* = 2 (A) measured using Evanno’s delta *K* and *K* = 4 (B) determined from the mean estimated Ln(*K*). More information found in Fig. S1.

The STRUCTURE analysis indicated that *K* = 2 and *K* = 4 are the most probable number of population clusters (Fig.2 and Fig. S1). The STRUCTURE plot based on *K* = 2 (Fig.2A) shows that all individuals are experiencing admixture but sites 33–35 (Zahrat Durakah, Abulad Island, Tiger Head, all from the Farasan Islands) are different from the rest of the dataset. The *K* = 4 plot (Fig.2B) again supports the uniqueness of sites 33–35, but it also supports separation of sites 30 and 36. To clarify, Site 30 (Wassalyat Shoals) forms one cluster, sites 33–35 (Zahrat Durakah, Abulad Island, Tiger Head, all from the Farasan Islands) form a second cluster, Site 36 (Socotra) forms a third cluster, and the rest of the dataset forms the fourth cluster.

The AMOVA indicates that the overall molecular variance among populations (36 sites) is low. When no regional classification of sites was used, the variation among populations was 4% (*P* = 0.001). When two regions were indicated (Region 1: sites 1–32 and Site 36 Socotra; Region 2: sites 33–35), the variation among populations was 3% (*P* = 0.001), while the variation among regions was 6% (*P* = 0.001). When a three-region classification was used (Region 1: sites 1–32; Region 2: sites 33–35; Region 3: Site 36 Socotra), the variation among populations was 2% (*P* = 0.001), while the variation among regions was 7% (*P* = 0.001). Likewise, when a four-region classification was used (Region 1: sites 1–29, 31–32; Region 2: Site 30; Region 3: sites 33–35; Region 4: Site 36), the variation among populations was 2% (*P* = 0.001), while the variation among regions was 7% (*P* = 0.001). In addition, there were many significant pairwise population comparisons with *F*_ST_ values ranging from 0 to 0.284 and *G*_ST_ values ranging from −0.011 to 0.077 (Table2). All pairwise comparisons with sites 30 (Mamali Kabir: Wassalyat Shoals), 33 (Farasan Islands: Zahrat Durakah), and 36 (Socotra: Socotra) were significant. Overall, the southern sites from the Farasan Banks, Farasan Islands, and Socotra were often significantly different from the rest of the dataset.

**Table 2:** Pairwise population structure based on nine^1^ microsatellite loci. Sites are indicated north to south (1–36). *F*_ST_ values are below the diagonal and *G*_ST_ values are above the diagonal. Values in bold show significant values based on FDR (alpha = 0.05)

	1	2	3	4	5	6	7	8	9	10	11	12	13	14	15	16	17	18	19	20	21	22	23	24	25	26	27	28	29	30	31	32	33	34	35	36
1		−0.006	0.005	−0.008	0.015	0.006	0.019	−0.004	0.014	**0.019**	0.003	0.005	0.006	0.002	0.008	0.012	0.015	**0.023**	0.012	0.006	0.018	**0.050**	0.011	0.008	0.009	**0.044**	**0.031**	**0.031**	**0.029**	**0.040**	0.014	0.016	**0.044**	**0.030**	**0.052**	**0.082**
2	0.000		−0.004	0.004	0.001	0.003	0.009	0.001	0.006	0.007	−0.001	**0.015**	0.002	0.001	0.003	0.003	−0.002	**0.015**	**0.014**	0.004	0.005	**0.027**	**0.010**	0.000	0.008	**0.021**	**0.025**	**0.020**	**0.011**	**0.027**	−0.005	−0.002	**0.042**	**0.023**	**0.045**	**0.074**
3	0.011	0.000		0.012	−0.004	0.001	0.015	0.004	0.003	−0.001	0.006	**0.026**	0.001	−0.002	0.004	0.001	−0.001	0.008	0.010	**0.012**	0.004	**0.020**	0.001	0.007	−0.011	**0.021**	**0.023**	**0.025**	0.006	**0.033**	−0.003	−0.003	**0.057**	**0.034**	**0.053**	**0.055**
4	0.000	0.009	0.023		0.010	0.010	**0.032**	0.002	0.008	**0.017**	−0.004	0.006	0.001	0.000	0.002	0.008	0.007	0.007	0.009	0.008	0.000	**0.035**	0.014	**0.021**	0.018	**0.027**	0.009	**0.025**	0.015	**0.021**	0.013	0.015	**0.032**	**0.018**	**0.040**	**0.044**
5	0.020	0.003	0.000	0.015		−0.004	0.013	0.007	**0.008**	0.006	0.004	**0.023**	0.002	0.008	**0.013**	0.006	0.003	**0.017**	**0.014**	**0.023**	**0.016**	**0.037**	**0.011**	0.007	0.008	**0.028**	**0.023**	**0.018**	**0.017**	**0.030**	0.006	0.002	**0.056**	**0.033**	**0.053**	**0.087**
6	0.015	0.007	0.001	0.021	0.000		0.006	0.001	0.006	0.005	0.004	**0.021**	0.005	0.006	**0.012**	0.008	**0.012**	0.013	**0.014**	**0.022**	0.013	**0.041**	**0.014**	0.004	0.008	**0.033**	**0.029**	**0.024**	**0.022**	**0.029**	0.008	0.001	**0.061**	**0.043**	**0.061**	**0.100**
7	0.025	0.006	0.022	0.036	0.026	0.013		0.011	**0.020**	**0.025**	**0.025**	**0.033**	**0.024**	**0.029**	**0.021**	**0.019**	**0.023**	**0.033**	**0.023**	**0.034**	**0.041**	**0.073**	**0.030**	0.007	**0.027**	**0.055**	**0.054**	**0.039**	**0.037**	**0.047**	0.007	0.011	**0.053**	**0.035**	**0.057**	**0.134**
8	0.000	0.003	0.008	0.002	0.015	0.002	0.012		0.007	**0.013**	0.001	0.011	0.002	0.008	0.002	0.008	0.008	0.014	0.006	0.006	0.013	**0.046**	**0.012**	−0.001	**0.018**	**0.031**	**0.017**	0.011	**0.017**	**0.032**	0.004	0.004	**0.044**	**0.027**	**0.042**	**0.080**
9	**0.025**	0.010	0.004	0.014	**0.015**	0.011	**0.026**	0.016		**0.009**	**0.007**	0.023	**0.005**	0.005	0.004	0.001	0.004	0.003	**0.007**	**0.013**	0.006	**0.031**	**0.009**	0.008	**0.012**	**0.017**	**0.016**	**0.010**	**0.014**	**0.022**	0.000	0.002	**0.047**	**0.027**	**0.047**	**0.062**
10	**0.033**	0.013	0.000	**0.032**	0.012	0.009	**0.040**	**0.029**	0.018		**0.011**	**0.025**	**0.007**	0.005	**0.012**	0.004	**0.008**	0.006	**0.017**	**0.019**	**0.008**	**0.025**	**0.008**	**0.015**	−0.003	**0.015**	**0.021**	**0.024**	**0.019**	**0.029**	0.007	0.002	**0.060**	**0.039**	**0.058**	**0.071**
11	0.000	0.000	0.008	0.000	0.007	0.006	**0.025**	0.001	**0.014**	**0.022**		0.003	0.002	0.005	0.004	0.008	0.006	**0.014**	**0.010**	**0.013**	0.007	**0.037**	**0.013**	**0.014**	**0.016**	**0.020**	**0.017**	**0.018**	**0.019**	**0.027**	0.004	0.008	**0.051**	**0.034**	**0.050**	**0.061**
12	0.012	**0.028**	**0.049**	0.014	**0.046**	**0.040**	**0.040**	**0.022**	**0.045**	**0.049**	0.007		**0.014**	**0.025**	**0.017**	**0.025**	**0.021**	**0.028**	**0.013**	**0.020**	**0.027**	**0.070**	**0.028**	**0.029**	**0.033**	**0.049**	**0.043**	**0.032**	**0.050**	**0.050**	**0.025**	**0.026**	**0.048**	**0.039**	**0.051**	**0.089**
13	0.005	0.003	0.002	0.000	0.006	0.010	**0.032**	0.005	**0.011**	**0.015**	0.004	**0.028**		0.002	**0.010**	0.004	0.001	**0.009**	**0.008**	**0.009**	**0.006**	**0.035**	**0.006**	0.007	0.007	**0.023**	**0.012**	**0.013**	**0.015**	**0.027**	0.002	0.003	**0.049**	**0.027**	**0.049**	**0.069**
14	0.004	0.002	0.000	0.000	**0.015**	0.012	**0.035**	0.015	0.009	**0.012**	0.009	**0.047**	0.004		0.000	−0.003	0.002	0.003	**0.008**	0.003	−0.002	0.014	0.002	0.010	−0.001	**0.015**	**0.011**	**0.016**	0.002	**0.016**	0.000	0.002	**0.040**	**0.022**	**0.038**	**0.045**
15	0.016	0.004	0.008	0.001	**0.026**	**0.025**	0.017	0.006	0.009	**0.026**	0.007	**0.033**	**0.021**	0.000		−0.003	0.005	0.004	**0.006**	0.004	0.003	**0.024**	**0.009**	0.006	**0.014**	**0.012**	**0.013**	**0.010**	0.004	**0.018**	−0.009	0.007	**0.037**	**0.022**	**0.032**	**0.039**
16	0.025	0.004	0.002	0.013	**0.014**	0.017	**0.025**	0.018	0.002	0.008	**0.013**	**0.048**	0.008	0.000	0.000		0.002	0.003	0.005	0.006	0.001	**0.023**	0.003	0.001	0.004	0.005	**0.011**	0.007	0.002	**0.016**	−0.009	−0.002	**0.047**	**0.026**	**0.044**	**0.062**
17	0.023	0.000	0.000	0.009	0.009	**0.021**	0.023	0.010	0.007	**0.015**	0.008	**0.035**	0.003	0.005	0.005	0.003		**0.011**	0.004	0.006	0.000	**0.034**	0.002	**0.012**	0.002	**0.019**	**0.019**	**0.019**	**0.010**	**0.028**	−0.003	−0.001	**0.050**	**0.026**	**0.050**	**0.059**
18	**0.031**	**0.028**	0.015	0.006	**0.029**	**0.022**	**0.045**	**0.025**	0.007	**0.014**	**0.025**	**0.052**	**0.016**	0.006	0.010	0.007	**0.019**		**0.013**	**0.012**	**0.007**	**0.018**	**0.008**	**0.015**	0.012	**0.016**	**0.011**	0.009	**0.012**	**0.019**	0.004	0.006	**0.053**	**0.031**	**0.047**	**0.053**
19	**0.027**	**0.024**	**0.018**	0.015	**0.030**	**0.028**	**0.027**	0.013	**0.014**	**0.034**	**0.019**	**0.025**	**0.017**	**0.014**	**0.012**	**0.010**	0.005	**0.026**		**0.011**	**0.010**	**0.054**	**0.007**	**0.015**	**0.017**	**0.033**	**0.023**	**0.012**	**0.021**	**0.029**	0.004	**0.010**	**0.048**	**0.030**	**0.049**	**0.066**
20	0.017	0.009	**0.024**	0.015	**0.045**	**0.043**	**0.040**	0.014	**0.027**	**0.039**	**0.026**	**0.040**	**0.021**	0.007	0.007	**0.011**	**0.010**	**0.025**	**0.020**		0.006	**0.034**	**0.010**	0.009	**0.015**	**0.024**	**0.021**	**0.017**	**0.013**	**0.031**	0.000	**0.010**	**0.032**	**0.018**	**0.029**	**0.054**
21	**0.034**	0.016	0.015	0.002	**0.033**	**0.029**	**0.059**	**0.027**	**0.016**	**0.025**	**0.019**	**0.060**	**0.019**	0.000	**0.012**	0.009	0.005	**0.018**	**0.023**	**0.014**		**0.021**	0.006	**0.015**	0.005	0.007	**0.012**	**0.021**	0.006	**0.021**	−0.001	0.005	**0.050**	**0.032**	**0.051**	**0.043**
22	**0.072**	**0.054**	**0.041**	**0.058**	**0.068**	**0.082**	**0.110**	**0.089**	**0.058**	**0.049**	**0.065**	**0.129**	**0.062**	**0.027**	**0.043**	**0.042**	**0.059**	**0.037**	**0.097**	**0.064**	**0.047**		**0.038**	**0.046**	0.021	**0.025**	**0.035**	**0.047**	**0.018**	**0.035**	0.015	**0.022**	**0.064**	**0.044**	**0.058**	**0.055**
23	**0.029**	**0.020**	0.004	**0.030**	**0.022**	**0.028**	**0.047**	**0.026**	**0.018**	**0.018**	**0.029**	**0.058**	**0.015**	0.005	**0.022**	0.008	0.007	**0.020**	**0.016**	**0.021**	**0.013**	**0.076**		**0.010**	−0.002	**0.017**	**0.020**	**0.016**	**0.013**	**0.033**	0.003	0.004	**0.064**	**0.039**	**0.063**	**0.062**
24	0.006	0.000	0.011	0.026	0.013	0.008	0.010	0.000	0.011	**0.025**	**0.017**	**0.048**	0.010	0.013	0.004	0.000	0.013	**0.021**	**0.024**	0.010	**0.026**	**0.073**	**0.018**		**0.016**	**0.023**	**0.024**	0.009	**0.014**	**0.030**	−0.006	0.003	**0.059**	**0.033**	**0.057**	**0.100**
25	0.022	0.018	0.000	**0.038**	0.017	0.015	**0.044**	**0.035**	**0.021**	0.000	**0.028**	**0.062**	0.012	0.000	**0.026**	0.009	0.004	**0.023**	**0.031**	**0.030**	**0.021**	**0.041**	0.000	**0.027**		**0.016**	**0.029**	**0.036**	**0.015**	**0.038**	0.006	0.000	**0.068**	**0.043**	**0.065**	**0.061**
26	**0.075**	**0.041**	**0.040**	**0.049**	**0.053**	**0.065**	**0.085**	**0.063**	**0.033**	**0.030**	**0.037**	**0.093**	**0.042**	**0.030**	**0.024**	0.009	0.035	**0.034**	**0.061**	**0.046**	**0.022**	**0.047**	**0.036**	**0.036**	**0.031**		**0.017**	**0.020**	**0.015**	**0.025**	0.006	**0.010**	**0.077**	**0.052**	**0.070**	**0.065**
27	**0.054**	**0.046**	**0.043**	0.015	**0.043**	**0.054**	**0.079**	**0.033**	**0.030**	**0.042**	**0.032**	**0.080**	**0.022**	**0.021**	**0.024**	0.019	0.030	**0.022**	**0.043**	**0.040**	**0.030**	**0.066**	**0.042**	**0.039**	**0.050**	**0.032**		**0.015**	**0.008**	**0.022**	0.009	**0.017**	**0.059**	**0.038**	**0.057**	**0.060**
28	**0.043**	**0.040**	**0.049**	**0.038**	**0.033**	**0.048**	**0.063**	0.021	**0.021**	**0.050**	**0.034**	**0.063**	**0.027**	**0.031**	**0.020**	0.016	0.032	**0.017**	**0.025**	**0.032**	**0.035**	**0.087**	**0.033**	0.014	**0.069**	**0.040**	**0.030**		**0.020**	**0.022**	0.009	**0.013**	**0.055**	**0.031**	**0.044**	**0.088**
29	**0.053**	**0.020**	0.010	**0.028**	**0.030**	**0.040**	**0.056**	**0.033**	**0.026**	**0.036**	**0.037**	**0.094**	**0.029**	0.005	**0.009**	0.003	**0.017**	**0.026**	**0.039**	**0.026**	**0.017**	**0.035**	**0.025**	**0.021**	**0.026**	**0.029**	**0.016**	**0.038**		**0.022**	−0.004	**0.009**	**0.056**	**0.033**	**0.052**	**0.047**
30	**0.069**	**0.051**	**0.060**	**0.037**	**0.054**	**0.055**	**0.071**	**0.064**	**0.043**	**0.056**	**0.051**	**0.095**	**0.052**	**0.032**	**0.036**	**0.030**	**0.049**	**0.038**	**0.056**	**0.061**	**0.048**	**0.067**	**0.065**	**0.051**	**0.069**	**0.048**	**0.043**	**0.044**	**0.042**		0.014	**0.024**	**0.058**	**0.031**	**0.056**	**0.078**
31	0.025	0.000	0.000	0.023	0.010	0.015	0.005	0.006	0.000	0.013	0.003	**0.046**	0.003	0.000	0.000	0.000	0.000	0.005	0.006	0.001	0.007	0.031	0.006	0.000	0.011	0.012	0.014	0.018	0.000	**0.024**		−0.010	**0.043**	**0.021**	**0.043**	**0.055**
32	0.025	0.000	0.000	0.023	0.005	0.002	0.011	0.006	0.005	0.005	**0.014**	**0.050**	**0.008**	0.003	**0.011**	0.000	0.000	**0.012**	**0.019**	**0.018**	0.009	**0.038**	**0.008**	0.003	0.001	**0.018**	**0.031**	**0.022**	**0.016**	**0.046**	0.000		**0.048**	**0.028**	**0.050**	**0.074**
33	**0.092**	**0.081**	**0.105**	**0.066**	**0.107**	**0.114**	**0.069**	**0.087**	**0.087**	**0.112**	**0.095**	**0.091**	**0.087**	**0.074**	**0.067**	**0.084**	**0.084**	**0.099**	**0.086**	**0.058**	**0.097**	**0.117**	**0.119**	**0.099**	**0.122**	**0.140**	**0.105**	**0.105**	**0.102**	**0.107**	**0.079**	**0.086**		0.001	0.002	**0.093**
34	**0.058**	**0.044**	**0.062**	**0.036**	**0.063**	**0.081**	**0.046**	**0.056**	**0.053**	**0.074**	**0.066**	**0.076**	**0.050**	**0.042**	**0.045**	**0.048**	**0.047**	**0.061**	**0.057**	**0.036**	**0.065**	**0.083**	**0.076**	**0.056**	**0.077**	**0.096**	**0.071**	**0.063**	**0.062**	**0.059**	**0.038**	**0.054**	0.004		**0.008**	**0.078**
35	**0.098**	**0.086**	**0.099**	**0.079**	**0.100**	**0.115**	**0.082**	**0.085**	**0.089**	**0.109**	**0.096**	**0.097**	**0.089**	**0.072**	**0.063**	**0.082**	**0.088**	**0.089**	**0.090**	**0.057**	**0.100**	**0.110**	**0.119**	**0.096**	**0.119**	**0.130**	**0.106**	**0.085**	**0.096**	**0.105**	**0.081**	**0.091**	0.006	**0.017**		**0.085**
36	**0.205**	**0.234**	**0.213**	**0.177**	**0.235**	**0.262**	**0.284**	**0.228**	**0.195**	**0.218**	**0.191**	**0.235**	**0.171**	**0.172**	**0.182**	**0.214**	**0.199**	**0.180**	**0.201**	**0.196**	**0.178**	**0.210**	**0.209**	**0.249**	**0.220**	**0.230**	**0.192**	**0.224**	**0.195**	**0.225**	**0.232**	**0.215**	**0.243**	**0.208**	**0.228**	

Values are calculated with 9999 permutations of the full dataset.

1 Only eight loci were used for calculating *F*_ST_ and *G*_ST_ for Site 36 as loci sc90 would not amplify for these samples.

### Geographic analysis

**Figure 3:**
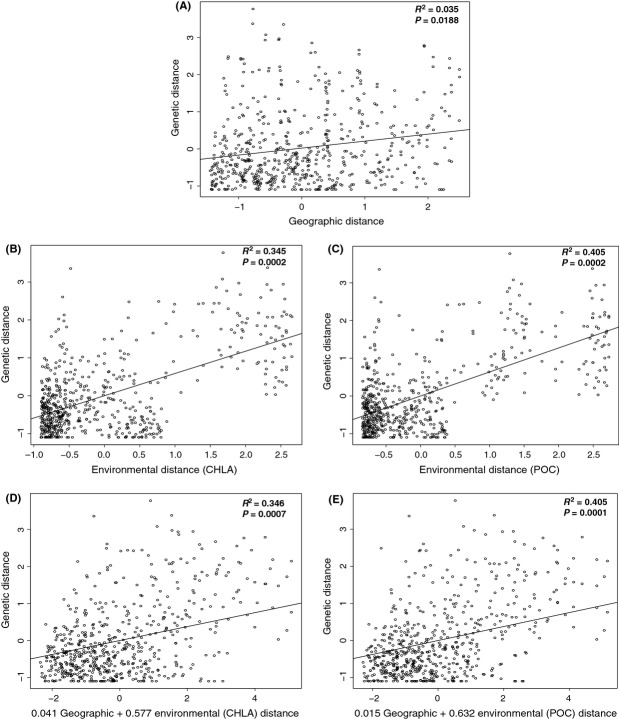
IBD and IBE analyses. The Mantel test scatterplot of IBD shows genetic distance (*F*_ST_ / (1 − *F*_ST_)) as a function of geographic distance (A) (Soccotra samples were withheld from all IBD and IBE analysis). The multiple matrix regression with randomization (MMRR) analyses shows IBE relationships between chlorophyll and genetic distance (B) and particulate organic matter and genetic distance (C) and for the combined effects of geographic and chlorophyll distances on genetic distance (D) as well as geographic and particulate organic matter distance on genetic distance (E). All data were standardized to a mean of zero and a standard deviation of one (unstandardized IBD plot can be seen in Figure S3).

Overall, the IBD Mantel tests show a weak positive (unstandardized matrices *R*^2^ = 0.0354, standardized matrices *R*^2^ = 0.035) but significant (unstandardized matrices *P* = 0.001, standardized matrices *P* = 0.0188) relationship between geographic distance and genetic distance (Fig.3A and Fig. S3), suggesting that geographic distance does not strongly influence genetic structure.

### Environmental analysis

The IBE Mantel tests comparing environmental distance (NSST, DSST, CHLA, POC, CDOM) with genetic distance (*F*_ST_ / (1 − *F*_ST_)) and geographic distance (GGD) yielded several significant correlations (*α *= 0.05). Geographic distance was significantly correlated with all environmental criteria, yet correlations with CHLA, POC, and CDOM were weak, *R*^2^ = 0.065, 0.075, and 0.031, respectively. Genetic distance was also strongly correlated with several of the environmental measurements, and these correlations were consistent for the two time frames (annual and winter) studied (Table3). The consistency in results suggests that there is no seasonal effect of environment on genetic structure, or at least that it is not detectable in this study. Genetic distance was significantly correlated with annual CHLA (*P* = 0.0000, *R*^2^ = 0.3446, *m* = 13.052), annual POC (*P* = 0.0000, *R*^2^ = 0.4045, *m* = 4839.3), winter CHLA (*P* = 0.0010, *R*^2^ = 0.3399, *m* = 12.784), and winter POC (*P* = 0.0000, *R*^2^ = 0.4070, *m* = 4927.5) (Table3).

**Table 3:** IBE Mantel test results of full dataset comparing genetic distance (Lin*F*_ST_) and geographic distance (GGD) with five environmental criteria gathered from 9-year averages of night sea surface temperature (NSST), day sea surface temperature (DSST), chlorophyll a (CHLA), particulate organic carbon (POC), and colored dissolved organic matter (CDOM) for the entire year (Annual) and October to May (Winter). *R*^2^ is indicated below the diagonal; *P* values are indicated above the diagonal. Calculations are based on 9999 permutations

	Lin*F*_ST_	GGD	NSST	DSST	CHLA	POC	CDOM
Annual							
Lin*F*_ST_		0.0190	0.1480	0.1200	0.0000	0.0000	0.2990
GGD	0.0353		0.0000	0.0000	0.0050	0.0040	0.0020
NSST	0.0110	0.8391		0.0000	0.1750	0.1320	0.0250
DSST	0.0149	0.8630	0.9821		0.1150	0.0990	0.0100
CHLA	0.3446	0.0650	0.0066	0.0161		0.0000	0.4230
POC	0.4045	0.0751	0.0112	0.0225	0.8630		0.3960
CDOM	0.0020	0.0306	0.0156	0.0222	0.0005	0.0008	
Winter							
Lin*F*_ST_		0.0190	0.1140	0.1680	0.0010	0.0000	0.2777
GGD	0.0353		0.0000	0.0000	0.0080	0.0040	0.3650
NSST	0.0121	0.8668		0.0000	0.1630	0.1110	0.4300
DSST	0.0069	0.8630	0.9860		0.2350	0.1690	0.4100
CHLA	0.3399	0.0615	0.0069	0.0028		0.0000	0.3260
POC	0.4070	0.0713	0.0137	0.0067	0.8556		0.2460
CDOM	0.0027	0.0002	0.0000	0.0000	0.0008	0.0026	

### Multiple matrix regression

Multiple matrix regression with randomization (MMRR) was used to determine whether ecological isolation (CHLA and POC) or geographic isolation is more likely contributing to the observed genetic structure in this study. The MMRR analysis showed that environmental distance (both CHLA and POC) is a stronger predictor of genetic distance than geographic distance (Fig.3) and in the combined models of geographic distance and environmental distance, geography contributes minimally to the strength of the model (Fig.3D–E). Overall, the best models for predicting genetic distance were the model employing only particulate organic carbon distance and the combined model of particulate organic carbon and geography (Fig.3C and E). These models accounted for 40% of the variation in genetic distance.

### Fine-scale structure

**Figure 4:**
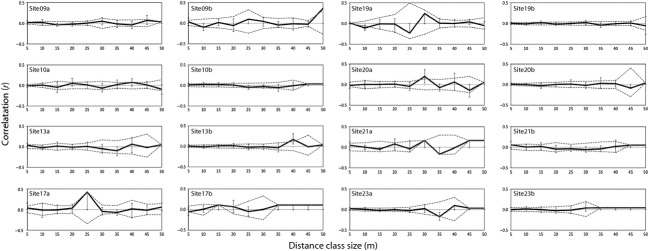
Small-scale spatial autocorrelation analysis for eight reef sites (9, 10, 13, 17, 19, 20, 21, 23) with two transects taken at each site. The relationship between geographic distance and genetic distance at the scale of 50 m is shown with correlograms. Distance class sizes of 5 m were chosen to highlight small spatial scales. The dashed gray lines represent 95% confidence intervals based on 999 permutations of individual locations among all individuals.

The spatial autocorrelation analysis of the 16 belt transects taken at 8 sites revealed no significant genetic autocorrelation at the scale of 50 m (Fig.4); that is, at this scale, the genotypes distribution seems to be random in all sites. In all analyses, the autocorrelation coefficient, r, was not significantly different from random expectations.

**Figure 5:**
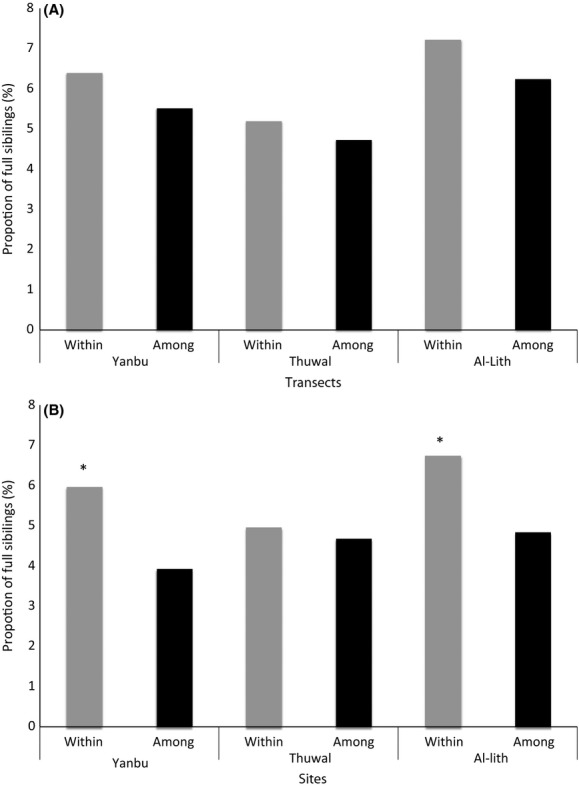
The proportion of significant full-sibling pairs within vs. among transects (A) and within vs. among sites (B). Significantly different proportions (*P* value < 0.05) of full-siblings are shown (*).

Overall, full-sibling pairs within transects, within sites, and among sites represented a small proportion of all pairwise comparisons. The proportion of full-sibling pairs ranged from 5.19% to 7.22% within transects, 4.72% to 6.24% among transects, 4.96% to 6.74% within sites, and 3.92% to 4.84% among sites (Fig.5). There was no significant difference (*P* > 0.05) between the proportion of full-siblings within transects vs. among transects at any of the three sampling locations (Yanbu χ^2^ = 0.68, df = 1, *P* = 0.20; Thuwal χ^2^ = 0.08, df = 1, *P* = 0.39; Al-Lith χ^2^ = 2.01, df = 1, *P* = 0.08; Fig.5A). There was, however, a significantly higher proportion of full-siblings within sites vs. among sites at Yanbu (χ^2^ = 10.29, df = 1, *P* = 0.00) and Al-Lith (χ^2^ = 29.32, df = 1, *P* = 0.00) (Fig.5B). This pattern was not seen at Thuwal; there was no significant difference between the proportion of full-siblings within and among sites (χ^2^ = 0.0593, df = 1, *P* = 0.4038). Overall, these results indicate that while at Thuwal individuals seem to be distributed randomly regardless of the scale, at Yanbu and Al-Lith, more related individuals are found at slightly larger than 50 m scales (100–200 m within sites) than is to be expected by chance (10–45 km among sites).

## Discussion

Overall, our broad-scale results suggest that environment is a better predictor of genetic structure of *S. carteri* than is geographic distance. Our fine-scale results indicate that at the scale of 50 m, genetic correlations are no different from random expectations, but at the scale of hundreds of meters, the results suggest that local dynamics might be important. Here we discuss the implications of our findings and propose hypotheses that could link these findings with biological processes that could be tested in future studies.

In terms of the broad-scale patterns that are seen in the data herein gathered, it seems that gene flow can be achieved throughout great distances in this species, at least in the north and central Red Sea. However, the southern region of the Red Sea, namely the Farasan Islands, seems genetically distinct from the rest of the Red Sea. Additionally in the Red Sea, there is evidence for restricted gene flow at Wassalyat Shoals, which lies in the transition zone between the Farasan Islands and the complex Farasan Banks (the southernmost area of the relatively genetically homogenous remainder of the Red Sea). Furthermore, in the northwest Indian Ocean, Socotra could represent another genetic cluster. The finding of strong genetic structure in the Farasan Islands is interesting in that it is similar to the results recently published in another study (Nanninga et al. 2014), yet Nanninga and colleagues found a genetic break in populations of an anemonefish, *Amphiprion bicinctus,* in the southern Red Sea at 19°N which is considerably further north than the genetic break found in this study (around 16°N). The reason for the difference in this exact location of this genetic break may be due to a difference in the biology of the two species (e.g., the active swimming and sensory capabilities of fish larvae or a wider environmental tolerance for one of the species), a question warranting further investigation. Regardless of the exact location of the boundary, it is notable that the southern Farasan Islands and potentially the Wassalyat Shoals of the Red Sea appear to host populations exhibiting this type of genetic break compared to the rest of the Red Sea. From this, we gather that there could be differential larval survival of *S. carteri* from the southern Red Sea. It is possible the nutrient poor waters found in the north and central Red Sea could present a boundary to larval survival for those individuals originating from the southern Red Sea (implying some level of local adaptation). And while in the past local adaptation was thought to be limited in marine invertebrates and restricted to only those species with very short dispersal distances, it has now been found that local adaptation occurs in many marine invertebrates, including those with planktonic dispersal (see review by Sanford and Kelly 2011). Further to this, a recent study of a sessile reef dweller has shown that strong selection on larvae produces locally adapted adults with narrow hybrid zones (Prada and Hellberg 2014).

The lack of a strong relationship between geographic distance and genetic distance in the IBD analysis indicates that long-range dispersal or stochastic colonization over many generations is allowing for a high degree of admixture within the Red Sea. It is possible that the inherent biological dispersal capability of *S. carteri* is most likely not a driving factor contributing to the genetic separation of the Farasan Islands from the rest of the Red Sea. But rather, it is hypothesized that gene flow is limited by other factors such as environmental heterogeneity or circulation boundaries. The IBE results indicate that environmental heterogeneity could be influencing gene flow. Most notably, the strong correlation between chlorophyll distance and genetic distance, and between particulate organic carbon distance and genetic distance indicate that differential primary productively might be associated with population divergence. However, further tests including transplant experiments are needed to fully determine whether or not the well-documented environmental gradient of highly oligotrophic waters in the north to central Red Sea and the more nutrient rich southern Red Sea (Raitsos et al. 2011, 2013, Table S2) are promoting local adaptation through either pre- or postrecruitment barriers to gene flow.

It is noted that the physical circulation of the Red Sea must be considered when determining barriers to gene flow in this region. The 9-year annual mean surface circulation model produced by Sofianos and Johns (2003) presents two notable circulation features in the southern Red Sea; these include the boundary current switch in the southern end of the Red Sea around 14.5°N and an anticyclonic gyre centered around 15–16°N. These physical features could create a pocket of isolation on the eastern side of the Red Sea, south of the boundary switch (14.5°N) that would be maintained by the turbulence effect of the eddy. However, the proposed pocket of isolation lies south of the southern Farasan Islands including sites 33–35. Because the sites in which we have detected barriers to gene flow lie north of this pocket where the model predicts a dominant northward flow north of 16°N, there is no large observable circulation feature that would promote isolation of sites 33–35. This suggests that circulation processes are not the only determining factor in this system. Overall, these observations are purely qualitative; thus, until the effect isolation by circulation is explicitly tested, and more sites in the southern and western Red Sea are studied, circulation cannot be ruled out as a predictor of genetic structure. But, the lack of a well-resolved Red Sea-wide circulation model and the difficulty of sampling reefs off the coast of Sudan, Yemen, and Somalia currently limit the ability to adequately address the influence of circulation in this system.

While we found no evidence for deviations from random mating at the scale of 50 m, the evidence for a greater proportion of full-siblings at scales of 100–200 m in *Stylissa carteri* assemblages suggests that dispersal might be limited to hundreds of meters or, alternatively, reproductive strategies in this organism might be complex. Besides potential dispersal limitation at scales slightly larger than 50 m, it is possible that low observed heterozygosity and high inbreeding coefficient (*F*_IS_) are the consequence, to some extent, of sporadic self-fertilization events. While the presence of null alleles cannot be completely ruled out, self-fertilization or asexual reproduction is relatively common in sponge populations and occurs in all classes within phylum Porifera (see review by Uriz and Turon 2012). It is possible that the reproductive mode of this sponge could be triggered by environmental fluctuations, or after environmental events that drastically diminish population densities. It is well documented that asexual reproduction can be favorable following habitat perturbation thereby allowing for rapid recolonization (Bell 1982; Trivers 1985). Though the extent to which this holds true for sponges is not well understood, it has, however, been shown in Antarctic sponges that asexual reproduction can allow for an increase in sponges with favorable genotypes in a relatively unchanging habitat (Carvalho 1994). Regardless of the known mechanism that might be promoting nonrandom mating, the lack of significant differences in the proportion of full-siblings within vs. among transects paired with the significantly greater proportion of full-siblings within sites vs. among sites indicates that there could be some site-specific structure at scales slightly larger than 50 m but less than the thousands of meters assessed with the among-site comparisons.

This study supports the growing body of literature supporting the importance of the environmental landscape in shaping the genetic structure of marine invertebrate populations (see review by Sanford and Kelly 2011); this study also represents the first evidence for a potential latitudinal environmental gradient influence on sponge population genetic structure. Besides providing important theoretical insights into sponge population dynamics, this study presents important information concerning the Red Sea ecosystem. This information is useful for the planning of marine protected areas by highlighting regions that appear to have frequent genetic exchange. In a broader biogeographic context, these results may help to clarify some of the underlying mechanisms that created or that maintain high endemism levels within the Red Sea.

## Data Accessibility

Genotypes: Dryad Data Repository doi: 10.5061/dryad.h2 h08. MMRR code: Dryad Data Repository doi: 10.5061/dryad.kt71r (Wang 2013).

## Supporting Information

**Table S1.** Primer multiplex mixes for nine microsatellite loci for *Stylissa carteri*. **Table S2.** Environmental data gathered for each of the 36 sample sites. **Table S3.** Summary of Multilocus Genotype matches for detection of clonal pairs in the dataset. **Table S4.** Tests for HWE for nine microsatellite loci *(sc58, sc65, sc72, sc88, sc90, sc56, sc83, sc82, sc96)* at 36 populations. **Figure S1.** STRUCTURE Harvester results indicate that *K* = 4 using the natural log of the maximum likelihood as score (A) and *K* = 2 using the using Evanno’s delta *K* (B) are the most probable number of theoretical data clusters. **Figure S2.** Large-scale population genetic differentiation as measured with Bayesian clustering via STRUCTURE (v2.3.4 Pritchard et al. 2000). Population prior was given; the number of clusters plotted is three (K = 3). **Figure S3.** Mantel test scatterplot of IBD shows genetic distance (*F*_ST_ / (1 − *F*_ST_)) as a function of geographic distance. **Figure S4.** Photograph of Stylissa carteri in the Red Sea (photograph credit: Tane Sinclair-Taylor).
