# Supplementary material for: Exploring seascape genetics and kinship in the reef sponge Stylissa carteri in the Red Sea
Source: Ecol Evol. 2015 Jun 1;5(13):2487–502. doi: 10.1002/ece3.1511 (PMC4523348; doi:10.1002/ece3.1511)

**Supporting Information:**

**Table S1** Primer multiplex mixes for nine microsatellite loci for *Stylissa carteri*

| Multiplex Mix | Locus (dye) | Primer sequence F and R (5'-3') | Ta (°C) | Repeat motif | Size range (bp) |
| --- | --- | --- | --- | --- | --- |
| Mix04 | sc58 (PET) | F:TGTGGCCTTCTGTCTATGCC | 57 | AC | 90-112 |
|  |  | R:CTAGCGTTTGACTCATGCCG |  |  |  |
|  | sc65 (VIC) | F:CGTAGAAGTCTCGCCCAAATG | 57 | AC | 184-232 |
|  |  | R:ACTCGGCCGTAAGTCATAGG |  |  |  |
| Mix06 | sc72 (VIC) | F:CATGGCATGCTTCACCTGAG | 60 | AC | 147-159 |
|  |  | R:TCAGTGCCGTTGTCCTCTTC |  |  |  |
|  | sc88 (6FAM) | F:AGTCAGGGATGTGGCTAGAG | 60 | AC | 190-224 |
|  |  | R:AGGAAGTAGCCAACGGAGAC |  |  |  |
| Mix07 | sc90 (6FAM) | F:CACACTCATGATCACACGCG | 60 | AC | 108-130 |
|  |  | R:ACATTCGCTACACTTGTGGG |  |  |  |
|  | sc56 (VIC) | F:CCATCTCACGATCCCTCTGG | 60 | AC | 122-136 |
|  |  | R:TCGGTGACCCTTCTTCTGTC |  |  |  |
| Mix09 | sc83 (NED) | F:GAACTGCAGGAAGGTGAGTG | 60 | AC | 173-189 |
|  |  | R:ACGTGAGGGAAGACAGTGAC |  |  |  |
| Mix10 | sc82 (VIC) | F:GTAGTTGGTGAGCGTGATGG | 63 | AC | 152-162 |
|  |  | R:TACCGCCTTCAAATGGCAAC |  |  |  |
|  | sc96 (6FAM) | F:AGTGTGCCCTTCCTCTCAAG | 63 | AC | 190-226 |
|  |  | R:ACCCTACAGTCACATGCACC |  |  |  |

**Table S2** Environmental data gathered for each of the 36 sample sites. Only 4 km^2^ data resolution was available thus, the degree difference in latitude *(Diff Lat)* and longitude *(Diff Long)* from the sample site and the data retrieval are given. Nine year averages for annual and winter values of night sea surface temperature *(nSST)*, day sea surface temperature *(dSST)*, chlorophyll concentration *(Chla)*, particulate organic carbon *(POC)*, and colored dissolved organic matter *(CDOM)* are given

| Site | Lat | Long | Diff Lat | Diff Lon | Winter  nSST | dSST | Chla | POC | CDOM | Annual  nSST | dSST | Chla | POC | CDOM |
| --- | --- | --- | --- | --- | --- | --- | --- | --- | --- | --- | --- | --- | --- | --- |
| 1 | 28.18 | 34.64 | 0.00 | 0.08 | 24.52 | 24.75 | 0.20 | 68.91 | 5.01 | 23.54 | 23.74 | 0.26 | 86.46 | 5.79 |
| 2 | 27.91 | 35.07 | 0.01 | 0.00 | 25.83 | 26.04 | 0.20 | 60.91 | 4.03 | 24.55 | 24.69 | 0.25 | 70.30 | 4.33 |
| 3 | 27.82 | 35.11 | 0.00 | 0.00 | 25.83 | 26.09 | 0.49 | 111.54 | 4.00 | 24.55 | 24.77 | 0.54 | 123.33 | 4.45 |
| 4 | 27.64 | 35.31 | 0.01 | 0.01 | 25.97 | 26.21 | 0.31 | 92.99 | 4.25 | 24.70 | 24.92 | 0.35 | 103.89 | 4.76 |
| 5 | 27.14 | 35.75 | 0.01 | 0.02 | 25.91 | 26.53 | 1.38 | 225.63 | 4.95 | 24.63 | 25.25 | 1.44 | 235.07 | 4.95 |
| 6 | 26.74 | 36.04 | 0.01 | 0.02 | 26.12 | 26.60 | 0.36 | 122.72 | 6.16 | 24.86 | 25.35 | 0.38 | 134.30 | 6.65 |
| 7 | 25.36 | 36.91 | 0.01 | 0.02 | 26.53 | 26.96 | 0.24 | 69.69 | 3.67 | 25.40 | 25.75 | 0.26 | 73.70 | 3.98 |
| 8 | 24.44 | 37.25 | 0.00 | 0.02 | 27.00 | 27.19 | 0.20 | 63.58 | 3.96 | 26.00 | 26.11 | 0.24 | 73.32 | 4.52 |
| 9 | 23.91 | 38.15 | 0.01 | 0.01 | 27.28 | 27.76 | 0.42 | 131.64 | 5.70 | 26.39 | 26.78 | 0.43 | 135.93 | 6.03 |
| 10 | 23.85 | 38.24 | 0.00 | 0.03 | 27.32 | 27.85 | 0.22 | 78.68 | 5.74 | 26.41 | 26.87 | 0.24 | 88.04 | 6.48 |
| 11 | 23.75 | 37.97 | 0.02 | 0.01 | 27.49 | 27.77 | 0.14 | 53.61 | 3.87 | 26.51 | 26.77 | 0.15 | 60.13 | 4.36 |
| 12 | 23.65 | 38.04 | 0.00 | 0.01 | 27.51 | 27.81 | 0.16 | 53.28 | 3.75 | 26.49 | 26.78 | 0.18 | 59.18 | 4.18 |
| 13 | 22.33 | 38.86 | 0.02 | 0.01 | 27.94 | 28.27 | 0.31 | 99.36 | 4.87 | 26.97 | 27.31 | 0.32 | 103.51 | 5.19 |
| 14 | 22.31 | 38.98 | 0.00 | 0.00 | 27.97 | 28.33 | 0.32 | 106.55 | 5.95 | 26.94 | 27.34 | 0.35 | 120.50 | 6.27 |
| 15 | 22.31 | 39.05 | 0.01 | 0.03 | 28.02 | 28.44 | 0.42 | 137.37 | 6.03 | 26.92 | 27.41 | 0.45 | 151.59 | 6.44 |
| 16 | 22.23 | 39.03 | 0.00 | 0.01 | 27.92 | 28.45 | 1.13 | 248.79 | 5.74 | 26.80 | 27.40 | 1.12 | 250.84 | 5.93 |
| 17 | 22.07 | 38.78 | 0.01 | 0.01 | 27.97 | 28.29 | 0.28 | 91.24 | 4.72 | 27.03 | 27.37 | 0.32 | 102.67 | 5.11 |
| 18 | 21.67 | 38.84 | 0.02 | 0.01 | 28.08 | 28.40 | 0.51 | 145.11 | 5.44 | 27.17 | 27.50 | 0.50 | 150.22 | 5.45 |
| 19 | 20.12 | 40.22 | 0.01 | 0.01 | 28.95 | 29.43 | 0.71 | 249.17 | 6.45 | 28.65 | 28.98 | 0.71 | 247.72 | 5.90 |
| 20 | 20.03 | 40.15 | 0.01 | 0.00 | 28.76 | 29.22 | 0.41 | 163.18 | 6.23 | 28.44 | 28.78 | 0.42 | 168.93 | 6.27 |
| 21 | 19.84 | 39.92 | 0.02 | 0.02 | 28.63 | 29.02 | 0.22 | 68.49 | 4.98 | 28.11 | 28.50 | 0.23 | 69.94 | 4.97 |
| 22 | 19.75 | 37.45 | 0.02 | 0.01 | 28.15 | 28.62 | 0.47 | 114.90 | 4.87 | 27.15 | 27.58 | 0.49 | 120.48 | 4.91 |
| 23 | 19.75 | 39.91 | 0.02 | 0.03 | 28.66 | 29.05 | 0.23 | 70.82 | 4.49 | 28.12 | 28.51 | 0.24 | 72.51 | 4.67 |
| 24 | 19.21 | 40.11 | 0.02 | 0.01 | 28.77 | 29.14 | 0.24 | 86.48 | 4.71 | 28.26 | 28.65 | 0.25 | 87.30 | 4.88 |
| 25 | 19.01 | 40.15 | 0.02 | 0.00 | 28.79 | 29.16 | 0.40 | 100.85 | 4.60 | 28.26 | 28.64 | 0.40 | 100.74 | 4.57 |
| 26 | 18.66 | 40.83 | 0.01 | 0.01 | 29.14 | 29.54 | 0.40 | 113.68 | 5.32 | 28.66 | 29.04 | 0.41 | 115.13 | 5.34 |
| 27 | 18.21 | 41.33 | 0.02 | 0.02 | 29.30 | 29.70 | 0.91 | 343.01 | 5.60 | 28.88 | 29.16 | 0.87 | 338.42 | 5.87 |
| 28 | 18.19 | 41.11 | 0.00 | 0.01 | 29.20 | 29.57 | 0.65 | 299.24 | 6.01 | 28.67 | 29.00 | 0.69 | 334.14 | 5.62 |
| 29 | 18.07 | 40.89 | 0.01 | 0.01 | 28.93 | 29.43 | 0.34 | 112.49 | 4.45 | 28.40 | 28.80 | 0.35 | 114.55 | 4.67 |
| 30 | 17.61 | 41.67 | 0.00 | 0.02 | 29.18 | 29.59 | 1.07 | 586.99 | 5.79 | 28.78 | 29.00 | 1.08 | 589.89 | 5.43 |
| 31 | 16.98 | 41.38 | 0.00 | 0.01 | 28.73 | 29.36 | 1.31 | 363.54 | 6.44 | 28.12 | 28.51 | 1.31 | 369.98 | 6.75 |
| 32 | 16.87 | 41.44 | 0.02 | 0.00 | 28.66 | 29.32 | 1.46 | 361.74 | 5.59 | 27.99 | 28.43 | 1.47 | 365.28 | 5.11 |
| 33 | 16.84 | 42.30 | 0.01 | 0.01 | 29.30 | 29.86 | 2.31 | 654.83 | 5.22 | 28.82 | 29.13 | 2.28 | 664.49 | 5.47 |
| 34 | 16.80 | 42.20 | 0.03 | 0.01 | 29.34 | 29.94 | 2.78 | 958.19 | 4.42 | 28.91 | 29.17 | 2.76 | 981.81 | 4.25 |
| 35 | 16.79 | 42.20 | 0.02 | 0.01 | 29.34 | 29.94 | 2.78 | 958.19 | 4.42 | 28.91 | 29.17 | 2.76 | 981.81 | 4.25 |
| 36 | 12.67 | 54.18 | 0.02 | 0.01 | 26.32 | 26.88 | 0.91 | 153.60 | 2.92 | 26.71 | 27.39 | 0.80 | 138.66 | 3.11 |

**Table S3** Summary of Multilocus Genotype matches for detection of clonal pairs in the dataset. Tests were made using Genalex. One individual of each clonal pair was removed (*) from the final dataset to avoid over representation of genotypes.

| Site | Sample | Multilocus Genotype |  |
| --- | --- | --- | --- |
| 34 | 84 | 110110228228149161190196116118122122173187152162224224g | * |
| 34 | 57 | 110110228228149161190196116118122122173187152162224224g |  |
| 6 | G04 | 94112228228149149196198116116130130185187156156222222g | * |
| 6 | G02 | 94112228228149149196198116116130130185187156156222222g |  |
| 27 | 286 | 94114228228149149196196112112130136181187162162222222g | * |
| 27 | 272 | 94114228228149149196196112112130136181187162162222222g |  |
| 32 | F10 | 9494226226149159190196112118130130183183152168222222g | * |
| 32 | F03 | 9494226226149159190196112118130130183183152168222222g |  |
| 30 | D02 | 9494228228149149190198116116130130181187152152222222g |  |
| 23 | 232 | 9494228228149149190198116116130130181187152152222222g |  |
| 22 | 336 | 9494230232149149198198112112122128181181156156208222g | * |
| 22 | 327 | 9494230232149149198198112112122128181181156156208222g |  |
| 6 | G07 | 106106228228141147200200112112128128158158181187156158222226g | * |
| 6 | G05 | 106106228228141147200200112112128128158158181187156158222226g |  |
| 8 | H11 | 11611622422414714719019011211613013014614618118715215200g | * |
| 8 | H03 | 11611622422414714719019011211613013014614618118715215200g |  |
| 19 | 479 | 104104228228149157192198114114130130146158185187152154222222g | * |
| 19 | 455 | 104104228228149157192198114114130130146158185187152154222222g |  |
| 35 | 428 | 110112228228149149190196112116124124146146173173156156222222g | * |
| 35 | 413 | 110112228228149149190196112116124124146146173173156156222222g |  |
| 23 | 237 | 94102192192149149198200116116130130146158183187156156222222g | * |
| 23 | 212 | 94102192192149149198200116116130130146158183187156156222222g |  |
| 29 | 400 | 94114192228157157190190116116130138158158173183152162222222g | * |
| 29 | 399 | 94114192228157157190190116116130138158158173183152162222222g |  |
| 23 | 229 | 9494184186149149190198116116130130154154187187162162222222g | * |
| 23 | 215 | 9494184186149149190198116116130130154154187187162162222222g |  |
| 10 | 601 | 9494192228149149192198116116130136158158183187154162222222g | * |
| 10 | 599 | 9494192228149149192198116116130136158158183187154162222222g |  |
| 28 | 159 | 9494228228149149190196114130130130156156181187156156222222g | * |
| 27 | 297 | 9494228228149149190196114130130130156156181187156156222222g |  |
| 23 | 264 | 9494228228149149198198114114124128146158187187162162222222g | * |
| 23 | 262 | 9494228228149149198198114114124128146158187187162162222222g |  |

**Table S4** Tests for HWE for nine microsatellite loci *(sc58, sc65, sc72, sc88, sc90, sc56, sc83, sc82, sc96)* at 36 populations. Tests were performed in GenePop with 10,000 permutations. P – values for deviation from HWE are corrected using FDR

| Site | sc58 |  | sc65 |  | sc72 |  | sc88 |  | sc90 |  | sc56 |  | sc83 |  | sc82 |  | sc96 |  |
| --- | --- | --- | --- | --- | --- | --- | --- | --- | --- | --- | --- | --- | --- | --- | --- | --- | --- | --- |
| 1 | 0.03 | NS | - | - | - | - | 0.36 | NS | 0.05 | NS | 0.18 | NS | 0.66 | NS | 0.03 | NS | - | - |
| 2 | 0.22 | NS | 0.11 | NS | 0.08 | NS | 0.96 | NS | 0.11 | NS | 0.10 | NS | 0.90 | NS | 0.24 | NS | - | - |
| 3 | 0.02 | S | 1.00 | NS | 0.05 | NS | 0.69 | NS | 0.10 | NS | 0.02 | S | 0.24 | NS | 0.17 | NS | - | - |
| 4 | 0.40 | NS | 1.00 | NS | 0.08 | NS | 0.01 | NS | 1.00 | NS | 0.00 | S | 0.73 | NS | 0.24 | NS | - | - |
| 5 | 0.40 | NS | 0.05 | NS | 0.01 | S | 0.96 | NS | 0.00 | S | 0.00 | S | 0.08 | NS | 0.18 | NS | - | - |
| 6 | 0.02 | S | 1.00 | NS | 0.41 | NS | 0.86 | NS | 0.01 | S | 0.01 | S | 0.88 | NS | 0.01 | S | 1.00 | NS |
| 7 | 0.02 | S | 0.20 | NS | 0.07 | NS | 0.79 | NS | 0.28 | NS | - | - | 0.99 | NS | 0.27 | NS | - | - |
| 8 | 0.15 | NS | 0.24 | NS | 0.11 | NS | 0.39 | NS | 0.30 | NS | 0.00 | S | 0.06 | NS | 0.02 | S | 0.02 | S |
| 9 | 0.00 | S | 0.01 | NS | 0.00 | S | 0.72 | NS | 0.00 | S | 0.07 | NS | 0.71 | NS | 0.00 | S | 0.10 | NS |
| 10 | 0.00 | S | 0.02 | NS | 0.12 | NS | 0.79 | NS | 0.00 | S | 0.02 | S | 0.15 | NS | 0.00 | S | 0.00 | S |
| 11 | 0.00 | S | 0.58 | NS | 0.05 | NS | 0.72 | NS | 0.01 | S | 0.00 | S | 0.02 | NS | 0.00 | S | 0.00 | S |
| 12 | 0.00 | S | 0.36 | NS | 1.00 | NS | 0.03 | NS | 0.00 | S | 0.00 | S | 0.04 | NS | 0.00 | S | 1.00 | NS |
| 13 | 0.00 | S | 0.00 | NS | 0.04 | NS | 0.01 | NS | 0.00 | S | 0.00 | S | 0.66 | NS | 0.00 | S | 0.00 | S |
| 14 | 0.04 | NS | 0.95 | NS | 0.34 | NS | 0.49 | NS | 0.00 | S | 0.07 | NS | 0.10 | NS | 0.00 | S | 0.02 | S |
| 15 | 0.13 | NS | 0.33 | NS | 0.00 | S | 0.01 | NS | 0.00 | S | 0.00 | S | 0.51 | NS | 0.00 | S | 0.00 | S |
| 16 | 0.00 | S | 0.16 | NS | 1.00 | NS | 0.03 | NS | 0.00 | S | 0.00 | S | 0.75 | NS | 0.20 | NS | 1.00 | NS |
| 17 | 0.02 | S | 0.89 | NS | 0.04 | NS | 0.64 | NS | 0.00 | S | 0.20 | NS | 0.78 | NS | 0.00 | S | - | - |
| 18 | 0.00 | S | 0.01 | NS | 0.10 | NS | 0.59 | NS | 0.12 | NS | 0.77 | NS | 0.29 | NS | 0.00 | S | 0.05 | NS |
| 19 | 0.00 | S | 0.83 | NS | 0.00 | S | 0.75 | NS | 0.00 | S | 0.00 | S | 0.00 | S | 0.00 | S | 0.00 | S |
| 20 | 0.00 | S | 0.27 | NS | 0.01 | S | 0.48 | NS | 0.00 | S | 0.00 | S | 0.72 | NS | 0.00 | S | 0.01 | S |
| 21 | 0.18 | NS | 0.01 | NS | 0.00 | S | 0.49 | NS | 0.00 | S | 0.00 | S | 0.35 | NS | 0.00 | S | 0.18 | NS |
| 22 | 1.00 | NS | 0.83 | NS | - | - | 0.06 | NS | 0.04 | NS | 0.94 | NS | 0.00 | S | 0.05 | NS | 0.28 | NS |
| 23 | 0.00 | S | 0.00 | NS | 0.00 | S | 0.04 | NS | 0.00 | S | 0.04 | NS | 0.12 | NS | 0.00 | S | 0.00 | S |
| 24 | 0.80 | NS | 0.01 | NS | 0.02 | S | 0.69 | NS | 0.11 | NS | 1.00 | NS | 0.97 | NS | 0.14 | NS | 0.10 | NS |
| 25 | 1.00 | NS | 1.00 | NS | - | - | 0.22 | NS | 0.00 | S | 0.00 | S | 0.04 | NS | 0.65 | NS | 1.00 | NS |
| 26 | 0.60 | NS | 0.09 | NS | 0.03 | NS | 0.95 | NS | 0.03 | NS | 0.05 | NS | 0.01 | NS | 0.00 | S | 0.01 | S |
| 27 | 0.01 | S | 0.23 | NS | 0.00 | S | 0.33 | NS | 0.00 | S | 0.03 | S | 0.21 | NS | 0.05 | NS | 0.02 | S |
| 28 | 0.24 | NS | 0.26 | NS | 0.01 | S | 0.69 | NS | 0.19 | NS | 0.01 | S | 0.28 | NS | 0.68 | NS | 1.00 | NS |
| 29 | 0.14 | NS | 0.32 | NS | 0.00 | S | 0.04 | NS | 0.00 | S | 0.72 | NS | 0.03 | NS | 0.00 | S | 0.00 | S |
| 30 | 0.00 | S | 0.49 | NS | 0.01 | S | 0.88 | NS | 0.11 | NS | 0.00 | S | 0.44 | NS | 0.00 | S | 0.00 | S |
| 31 | 0.20 | NS | 0.14 | NS | - | - | 0.52 | NS | 1.00 | NS | 0.07 | NS | 0.82 | NS | 1.00 | NS | 0.06 | NS |
| 32 | 0.00 | S | 0.01 | NS | 0.00 | S | 0.51 | NS | 0.29 | NS | 0.00 | S | 0.04 | NS | 0.01 | S | 0.02 | S |
| 33 | 0.02 | S | 1.00 | NS | 0.02 | S | 0.82 | NS | 0.32 | NS | 0.01 | S | 0.08 | NS | 0.03 | NS | 0.13 | NS |
| 34 | 0.00 | S | 0.03 | NS | 0.09 | NS | 0.15 | NS | 0.00 | S | 0.00 | S | 0.34 | NS | 0.10 | NS | 0.00 | S |
| 35 | 0.14 | NS | 0.42 | NS | 0.05 | NS | 0.24 | NS | 0.00 | S | 0.24 | NS | 0.16 | NS | 0.00 | S | 1.00 | NS |
| 36 | - | - | 0.87 | NS | 0.04 | NS | 0.00 | NS | - | - | 0.60 | NS | 0.65 | NS | 0.85 | NS | 1.00 | NS |

**Fig S1** STRUCTURE Harvester results indicate that K = 4 using the natural log of the maximum likelihood as score *(a)* and K = 2 using the using Evanno’s delta K *(b)* are the most probable number of theoretical data clusters.

**Fig S2** Large-scale population genetic differentiation as measured with Bayesian clustering via STRUCTURE (v2.3.4 Pritchard et al. 2000). Population prior was given; the number of clusters plotted is three (K = 3).

Sites are listed along the x-axis; the likelihood score is listed on the y-axis.

Fig S3 Mantel test scatterplot of IBD shows genetic distance (F_ST_/(1- F_ST_)) as a function of geographic distance. Matrices were not standardized.

Fig S4 Photograph of Stylissa carteri in the Red Sea (photograph credit: Tane Sinclair-Taylor).
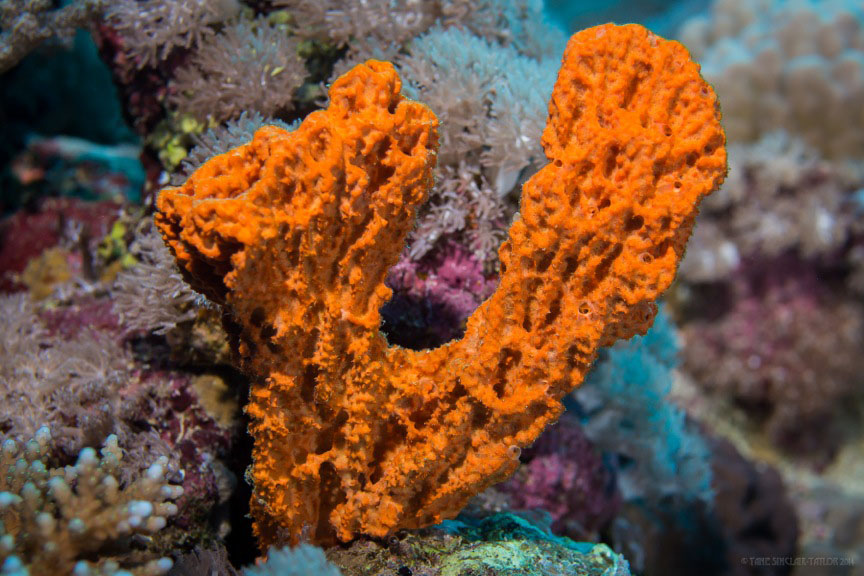

Supplement: Supplementary file 1 [file ece30005-2487-sd1.docx]
